# Factors associated with vaccination completion and retention among HIV negative female sex workers enrolled in a simulated vaccine efficacy trial in Kampala, Uganda

**DOI:** 10.1186/s12879-019-4328-1

**Published:** 2019-08-16

**Authors:** Yunia Mayanja, Andrew Abaasa, Gertrude Namale, Gershim Asiki, Matthew A. Price, Anatoli Kamali

**Affiliations:** 1 MRC/UVRI & LSHTM Uganda Research Unit, Plot 51-59 Nakiwogo Road, P. O Box 49, Entebbe, Uganda; 2 London School of Hygiene and Tropical Medicine, Keppel Street, London, WC1E 7HT UK; 3 African Population and Health Research Center, P.O. Box 10787-00100, Nairobi, Kenya; 4 Department of Women’s and Children’s Health, Karolinska Institutet, Tomtebodavägen 18a, 171 77 Stockholm, Sweden; 5 International AIDS Vaccine Initiative, 125 Broad St, New York, NY 10004 USA; 6 Department of Epidemiology and Biostatistics, University of California San Francisco, 550 16th Street, San Francisco, CA 94143 USA

**Keywords:** Female sex workers, Vaccination completion, HIV prevention trials, Retention, Sub Saharan Africa

## Abstract

**Background:**

Female sex workers (FSWs) at substantial risk of HIV are potentially a suitable group for HIV prevention trials including vaccine trials. Few HIV vaccine preparatory studies have been conducted among FSWs in Sub-Saharan Africa (SSA); data are therefore limited on acceptability of vaccine trial procedures. We determined vaccination completion and one-year retention among FSWs in Kampala, Uganda.

**Methods:**

We conducted a prospective study that simulated a vaccine efficacy trial among HIV negative FSWs (18–49 years). Hepatitis B vaccine (Engerix B) was used to mimic an HIV vaccine product. Volunteers received 1 ml intramuscular injection at 0, 1 and 6 months, and made additional visits (3 days post-vaccination and months 3, 9 and 12). They were censored at that visit if diagnosed as HIV positive or pregnant. We collected socio-demographic, behavioral and clinical data at baseline, 6 and 12 months and fitted Poisson regression models with robust standard error to find factors associated with vaccination completion and retention.

**Results:**

We enrolled 290 volunteers (median age 27 years) of whom 230 reached a study end-point as follows: 7 became HIV infected, 11 became pregnant and 212 completed both the vaccination schedule and 12-month visit giving a retention of 77.9% (212/272). Vaccination completion was 82.4%.

Non-retention at 1 year was more likely among those reporting symptoms of genital ulcer disease (GUD) in the past 3 months (IRR 1.90; 95% CI 1.09–3.32) and those < 35 years; (IRR 6.59; 95% CI 2.11–20.57). Non-completion of the vaccination schedule was associated with being < 35 years (IRR 13.10; 95% CI 1.89–90.92, reporting GUD symptoms (IRR 3.02; 95% CI 1.71–5.33) and reporting consistent condom use with new sexual partners (IRR 2.57; 95% CI 1.10–6.07).

**Conclusions:**

FSWs are at substantial risk of HIV infection and yet willing to participate in HIV vaccine and prevention research; young FSWs should be empowered, and those reporting GUD symptoms need close follow up to improve participation in future HIV vaccine trials.

## Background

To date, several HIV-1 vaccine candidates have gone through early phase clinical trials and shown acceptable safety profiles [1, 2]. However, fewer vaccine candidates have proceeded to phase III efficacy trials [1, 3], the RV144 trial conducted in Thailand being the first HIV vaccine efficacy trial to provide evidence that a preventative HIV vaccine is possible [4, 5]. Although the 31% efficacy achieved 3.5 years after vaccination was insufficient for licensure, it gave an opportunity to understand the immune correlates of protection and improve this vaccine. The RV144 regimen was then modified and assessed for safety and immunogenicity in the HVTN100 trial in South Africa. The modified vaccine in HIVTN100 elicited sufficiently high immune responses and, pre-specified immunological go/no-go criteria were achieved leading to initiation of an efficacy trial (HVTN702) [3, 6]. Several countries in Sub-Saharan Africa (SSA) are now following volunteers in the HVTN702 trial, assessing efficacy of one prime-boost regimen and adjuvanted recombinant envelope protein and HVTN705, assessing a mosaic adenovirus and clade C envelope prime-boost regimen. With the on-going HPTN084 trial comparing efficacy of long acting injectable cabotegravir and oral PrEP (Tenofovir Disoproxyl Fumarate /Emtricitabine), it is evident that several biomedical HIV prevention products may soon be available; it is for this reason that researchers in the International AIDS Vaccine Initiative (IAVI) network in SSA are preparing suitable populations for future HIV prevention efficacy trials of interventions such as long acting anti-retroviral drugs, protective antibodies and vaccines.

HIV vaccine preparatory studies to determine suitability of populations, and inform recruitment and retention strategies for future efficacy trials in SSA have followed up cohorts of fishing communities, men who have sex with men (MSM) and female sex workers (FSWs) where they demonstrated retention rates of ≥80% [7, 8]. In addition, studies of other HIV prevention interventions e.g. the ‘Partners PrEP trial which randomized discordant couples to oral pre-exposure prophylaxis (PrEP) and Placebo, and the Dapivirine ring study, which randomized high-risk women to the dapivirine ring and placebo, both showed efficacy of investigational products and retention of ≥85% [9, 10]. In the HIV vaccine field, preparedness studies that go beyond following up cohorts to conduct simulated vaccine efficacy trial (SiVET) procedures will inform acceptability of HIV vaccine research and retention among key populations where the need for a vaccine remains acute.

Key populations have a high HIV incidence and prevalence [11, 12], making them suitable potential volunteers in future vaccine efficacy trials. In SSA however there is still limited data on SiVETs among key populations such as FSWs whose social contexts may differ by region. In Uganda for example, social structures include women without capital who depend entirely on sex work for their livelihood, those engaging in an institutionalized trade often mediated by middlemen in entertainment facilities, and the minority who are more financially independent and earn from their own businesses as well as from sex work [13]. In Uganda like many parts of SSA, sex work is still criminalized and often times, the women encounter police brutality, stigmatization from their communities, and violence which is perpetrated by both clients and police [14, 15]. The women are also highly mobile [16] and, coupled with the criminalization and discrimination they face [17, 18], it is important to learn more about feasibility of enrolling FSWs in Uganda, and if they would comply with frequent study visits and vaccination procedures.

We conducted a SiVET using hepatitis B vaccine (Engerix B) as a proxy for an HIV vaccine to assess volunteer retention and completion of a three-injection vaccination schedule among FSWs in Kampala, Uganda. The study also provided an opportunity to develop and strengthen site operational tools such as standard operating procedures and quality management systems that will be useful in the successful conduct of large efficacy trials.

## Methods

### Study design and procedures

We enrolled volunteers into a prospective study designed to mimic the rigors of an HIV vaccine trial and administered a licensed recombinant Hepatitis B vaccine (ENGERIX-B™ GlaxoSmitheKline Biologicals Rixensart, Belgium). We used Hepatitis B vaccine because it shares some features with a potential HIV vaccine: the hepatitis B vaccination schedule is similar to that of proposed HIV vaccines and education messages to volunteers would be similar given that Hepatitis B and HIV are both chronic viral infections that share transmission modes. Hepatitis B was also of potential benefit to the volunteers as they are also at high risk for Hepatitis B infection. We enrolled volunteers between August 2014 and May 2016, and each was scheduled to attend 9 visits during a 12 month follow up period. At months 0, 1 and 6 they were scheduled to receive 1 ml of hepatitis B vaccine by intramuscular injection in the deltoid muscle of the non-dominant arm, and have two reactogenicity assessments: at least 30 min and 3 days after each vaccination. Trained study nurses performed baseline clinical assessments (including medical history and physical examination) before giving the first vaccination dose and administered questionnaires to collect study data. The study pharmacist dispensed Hepatitis B vaccine to study nurses who returned all used vials to the pharmacy. Vaccine accountability logs were used to document and monitor vaccine use. The vaccine was stored in a 2–8^0^ C refrigerator and vaccine safety was ensured by monitoring the cold chain daily and assessing volunteers for adverse events. Other study visits were scheduled at months 3, 9 and 12 at which volunteers received HIV testing and counselling (HTC), contraceptive services, syndromic management of sexually transmitted infections (STIs) and free health care for common illnesses. The SiVET visits had scheduled windows as follows: vaccination visits (±3 days), 3-day post vaccination visits (±1 day), all other visits (±7 days) as shown below.

SiVET study visit schedule

**Table:** 

	Visit 1	Visit 2	Visit 3	Visit 4	Visit 5	Visit 6	Visit 7	Visit 8	Visit 9
**Visit Month (Study Month = 28 days)**	**M0**		**M 1**		**M 3**	**M 6**		**M 9**	**M 12**
**Visit Window (Days)**		± **1**	± **3**	± **1**	± **7**	± **3**	± **1**	± **7**	± **7**
**Vaccination Visit**	**X**		**X**			**X**			
**3-day post vaccination**		**X**		**X**			**X**		

### Study population and sampling

We conducted the study at the Good Health for Women Project (GHWP) clinic of MRC/UVRI and LSHTM Uganda Research Unit, located in a peri-urban community in southern Kampala. Field workers conducted mobilization activities with community peer leaders to identify FSWs from commercial hotspots who were then enrolled at the clinic irrespective of HIV status as has been described by Vandepitte et al. [19]. They attended quarterly follow up visits to receive HIV prevention and treatment services which include HIV testing and counselling (HTC); syndromic management for STIs; contraception and, in addition, HIV care and treatment for HIV positive volunteers.

#### Sample size determination

We estimated one-year retention in a simulated vaccine efficacy trial to be 75% with a precision of ±5%. Recruiting 290 participants was expected to provide 80% power, at the 5% level of significance (two-sided).

We consecutively enrolled consenting HIV negative volunteers who had been attending the GHWP cohort for 6 to 18 months and were ≥ 18 years, sexually active in the 3 months before enrolment, not pregnant, willing to use effective contraception until 3 months after the last vaccination and not known to be allergic to yeast.

Volunteers were withdrawn from the study if they became either HIV positive or pregnant, were no longer interested in participating or were lost to follow up (LTFU). Those who became HIV infected or pregnant were censored at that study visit (i.e., taken off study to complete their hepatitis B vaccine schedule outside the study and receive further health care and treatment provided at the GHWP clinic or referred as appropriate). We defined LTFU as a volunteer not attending study visits for six consecutive months, and was not known to have died, moved out of the study area or withdrawn from the study.

### Volunteer retention

The study field team used phone calls to remind volunteers about follow up visits and for those who needed help to access the clinic, we used a project vehicle to pick them up. Study volunteers received reimbursement of 4USD for time and transport costs when they attended study visits. Free treatment for common illnesses and/ or referral for those who needed specialized health care were provided as needed and the study nurses always informed them about their next scheduled date, which was documented on their appointment card. Field workers kept track of volunteers’ visits using a visit tracker and volunteer visit calendars. When a volunteer missed one visit, the field workers traced her and encouraged her to come to the clinic for the next scheduled visit.

### Laboratory methods

HIV testing was performed on serum using two or more rapid antibody diagnostic tests administered serially as follows: Determine screening test (Alere Medical Co. Ltd., Chuba, Japan), Statpak rapid confirmatory kit (Chembo Diagnostics Systsem Inc., Medford, NY, USA) and Unigold as tie breaker (Trinity Biotech Plc, Wicklow, Ireland). Pregnancy tests were performed on urine using QuickVue tests strips (Quidel Corporation, San Diego CA, USA).

### Data collection

All data were collected using pre-designed interviewer administered questionnaires.

The primary study outcomes for this manuscript were vaccination completion and retention. We defined vaccination completion as a volunteer receiving all three doses of Hepatitis B vaccine and documented it as a binary outcome *(Yes/No)*.

Retention was defined as attendance of the month 12 visit by those who completed the 3-dose vaccine regimen, did not become HIV infected and did not become pregnant during the study. Volunteers who attended the visit outside the scheduled window were included and we documented retention as a binary outcome (*Yes/No*).

We selected independent variables based on literature review of multi-dose vaccine studies that assessed vaccination completion and/ or retention, and also included socio-demographic variables as potential confounders. The independent variables were as follows: Socio-demographic characteristics; age, education level and marital status.

Behavioral characteristics: alcohol use in the past month *(none, once a week or less and daily)*; being drunk before sex in the past month *(none, sometimes/ most times and always)*; drug use in the past month *(Yes/ No)*; new sexual partner(s) in the past 3 months *(Yes /No)*; condom use with new sexual partner(s) *(Yes/ No)*; receiving payment for sex in the past 3 months *(Yes/ No)*; giving payment for sex in the past 3 months *(Yes/ No).* Payment for sex was in form of money, gifts or favors.

Clinical characteristics: reported vaginal discharge syndrome (VDS) in the past 3 months *(Yes/ No)*, reported genital ulcer disease in the past 3 months *(Yes/ No)*.

### Statistical methods

The study data was double entered in OpenClinica (version 3.1, USA) and analyzed in STATA 14 (StataCorp, College Station, TX, USA). We summarized volunteer socio demographic and behavioral characteristics at baseline by counts and percentages. We estimated the proportion of volunteers that completed the entire vaccination regimen (i.e. all three vaccination visits) as number completing all vaccinations divided by the total number of volunteers enrolled. We further estimated the proportion of volunteers that were retained in the study as the number of volunteers that completed all three vaccinations and also attended month 12 visit divided by the total number that were expected to attend (those who received all 3 vaccination doses and were not censored between month 6 and month 12. The two proportions were stratified by the volunteer baseline characteristics. Data for each volunteer was divided into observation time points corresponding to scheduled clinic visits. For each outcome i.e. vaccination completion and retention, we fitted bivariable Poisson regression models with time-varying covariates to allow for intra-individual correlation (because individuals had multiple records) by using robust standard errors. After bivariable analyses, two multivariable models were fitted (i) for retention and (ii) for vaccination completion. Only factors for which the association attained a statistical significance at the 20% level using a likelihood ratio test in a bivariate analysis were considered for the multivariable model. In the multivariable model, factors were removed from the model using a backward elimination algorithm if removing the term did not make the fit of the model significantly worse at the 5% level on a likelihood ratio test.

## Results

### Baseline characteristics of FSWs enrolled in the SiVET study in Kampala, Uganda

We screened 381 volunteers for the study of whom 304 (79.8%) were eligible and 290 (76.1%) enrolled. The common reasons for not enrolling if eligible were returning late for the enrolment visit (8) and volunteers declining enrolment because they thought blood draws were too frequent (4). The median age of study volunteers was 27 years (range 18–56 years), 94.5% had attained primary level education or higher and 70.3% were either separated or widowed. New sexual partners in the past 3 months were reported by 94.5% of volunteers of whom 79.9% reported always using condoms with new partners. All except one reported receiving money, gifts or favors in exchange for sex while 16.2% also reported giving money, gifts or favors in exchange for sex in the past 3 months. Vaginal discharge syndrome (VDS) and genital ulcer disease (GUD) were reported by 29.7 and 18.6% respectively. Table 1 shows volunteer baseline characteristics stratified by study outcomes.

**Table 1:** Baseline Characteristics of FSWs Enrolled in a simulated vaccine Efficacy Trial, Kampala Uganda (2014–2016)

Variable	Sub Category	Total n (%)	Completed All Vaccinations n (%)	Completed All Vaccinations and Month 12 visit n (%)
Overall		290 (100)	239 (82.4)	212 (73.1)
Age (Yrs)	35+	62 (21.4)	58 (93.6)	55 (88.7)
	18–34	228 (78.6)	181 (79.4)	157 (68.9)
Education	None	16 (5.5)	12 (75.0)	10 (62.5)
	Primary	149 (51.4)	123 (82.6)	112 (75.2)
	Secondary or higher	125 (43.1)	104 (83.2)	90 (72.0)
Marital Status	Single never married	68 (23.4)	58 (85.3)	52 (76.5)
	Married	18 (6.2)	14 (77.8)	13 (72.2)
	Separated or widowed	204 (70.3)	167 (81.9)	147 (72.1)
Alcohol use in the past month	None	57 (19.7)	44 (77.2)	41 (71.9)
	≤ once a week	148 (51.0)	128 (86.5)	110 (74.3)
	Daily	85 (29.3)	67 (78.8)	61 (71.8)
Drug use in the past month	No	213 (73.4)	177 (83.1)	159 (74.7)
	Yes	77 (26.6)	62 (80.5)	53 (68.8)
Drunk before sex in the past month	Never	103 (35.5)	86 (83.5)	83 (80.6)
	Sometimes/ most times	170 (58.6)	140 (82.4)	119 (70.0)
	Always	17 (5.9)	13 (76.5)	10 (58.8)
Reported VDS in past 3 months	No	204 (70.3)	174 (85.3)	154 (75.5)
	Yes	86 (29.7)	65 (75.6)	58 (67.4)
Reported GUD in past 3 months	No	236 (81.4)	200 (84.8)	179 (75.9)
	Yes	54 (18.6)	39 (72.2)	33 (61.1)
Number of Sexual partners in past 3 months	0–2	14 (4.9)	11 (78.6)	10 (71.4)
	0–3	61 (21.0)	53 (86.9)	50 (82.0)
	≥ 6	215 (74.1)	175 (81.4)	152 (70.7)
New sexual partner in past 3 months	No	16 (5.5)	12 (75.0)	10 (62.5)
	Yes	274 (94.5)	227 (82.9)	202 (73.7)
Condom use with new partner(s)	Never	3 (1.1)	3 (100)	3 (100)
	Sometimes/ most times	52 (19.0)	48 (92.3)	42 (80.8)
	Always	219 (79.9)	176 (80.4)	157 (71.7)
Receiving payment for sex in past 3 months	No	1 (0.3)	1 (100)	1 (100)
	Yes	289 (99.7)	238 (82.4)	211 (73.0)
Giving payment for sex in past 3 months	No	243 (83.8)	202 (83.1)	181 (74.5)
	Yes	47 (16.2)	37 (78.7)	31 (66.0)

### Factors associated with missing any vaccination visit and drop out

Of the 290 enrolled women, 239 (82.4%) completed their 3-dose vaccine schedule, and 212 (73.1%) of these also attended the 12-month study visit. Nine women who did not complete their vaccination schedule also attended their 12-month study visit; these were not included in our final estimate of retention. In total, 230 reached a study end-point as follows: 7 became HIV infected, 11 became pregnant and 212 completed both the 3-dose vaccination schedule and the last visit at month 12 giving a retention of 77.9% (212/272).

At adjusted analysis, volunteers who dropped out by 12 months were more likely to report GUD symptoms in the past 3 months (IRR 1.90; 95% CI 1.09–3.32) and be < 35 years (IRR 6.59; 95% CI 2.11–20.57).

At adjusted analysis, missing any vaccination visit was associated with being < 35 years (IRR 13.10; 95% CI 1.89–90.92), reporting GUD symptoms in the past 3 months (IRR 3.02; 95% CI 1.71–5.33) and reporting consistent condom use with new sexual partners (IRR 2.57; 95% CI 1.10–6.07). We did not find education level, marital status and substance use (alcohol and illicit drugs) to be associated with either vaccination completion or retention. Details are in Table 2.

**Table 2:** Factors associated with missing any one-vaccination visit, and non-retention in the SiVET study

Variable	Sub Category	Missing any one vaccination visit			Non retention		
		Unadjusted IRR (95%CI)	LRT-*p* value	Adjusted IRR (95%CI)	Unadjusted IRR (95%CI)	LRT-*p* value	Adjusted IRR (95%CI)
Age (years)	35+	1.00	**0.013**	1.00	1.00	**0.002**	1.00
	18–34	**3.59 (1.31–9.83)**		**13.10 (1.89–90.92)**	**3.24 (1.52–6.88)**		**6.59 (2.11–20.57)**
Education level	None	1.00	0.666		1.00	0.492	
	Primary	0.67 (0.24–1.86)			0.61 (0.27–1.37)		
	Secondary or higher	0.62 (0.22–1.74)			0.67 (0.30–1.50)		
Marital Status	Single never married	1.00	0.669		1.0	0.714	
	Married	1.62 (0.53–4.93)			1.32 (0.51–3.40)		
	Separated or widowed	1.27 (0.63–2.54)			1.24 (0.72–2.12)		
Alcohol use in the past month	None	1.00	0.226		1.00	0.327	
	Once a week or less	0.59 (0.31–1.14)			0.73 (0.42–1.25)		
	Daily	0.91 (0.46–1.78)			1.02 (0.58–1.80)		
Drug use in the past month	No	1.00	0.841		1.00	0.295	
	Yes	1.06 (0.58–1.94)			1.28 (0.80–2.05)		
Drunk before sex in past month	Never	1.00	0.903		1.00	0.973	
	Sometimes /most times	0.98 (0.56–1.71)			1.03 (0.66–1.61)		
	Always	0.72 (0.17–2.99)			0.91 (0.29–2.87)		
Reported VDS in past 3 months	No	1.00	0.217		1.00	0.998	
	Yes	1.42 (0.81–2.45)			0.99 (0.62–1.62)		
Reported GUD in past 3 months	No	1.00	0.003	1.00	1.00	**0.012**	1.00
	Yes	**2.44 (1.36–4.37)**		**3.02 (1.71–5.33)**	1.58 (0.93–2.68)		**1.90 (1.09–3.32)**
Number of sexual partners	0–2	1.00	0.866		1.00	0.427	
	3–5	1.04 (0.31–3.53)			0.71 (0.29–1.72)		
	6+	1.22 (0.40–3.71)			1.06 (0.51–2.21)		
New sexual partner in past 3 months	No	1.00	0.481		1.00	0.268	
	Yes	0.73 (0.31–1.74)			0.71 (0.38–1.31)		
Condom use with new partner(s)	Never/Inconsistently	1.00	**0.03**	1.00	1.00	0.060	1.00
	Consistently	2.06 (0.82–5.20)		**2.57 (1.10–6.07)**	2.08 (0.97–4.47)		2.04 (1.00–4.15)
Giving payment for sex in past 3 months	No	1.00	0.380		1.00	0.204	
	Yes	1.35 (0.69–2.63)			1.42 (0.83–2.42)		

Multivariable analysis adjusted for age, genital ulcer disease in the past 3 months and condom use with a new partner(s)

Data in bold are statistically significant

## Discussion

In this cohort study designed to mimic the rigors of an HIV vaccine trial, retention (completion of the 3-dose vaccination schedule and the last study visit) was 78%. Our study is among the few SiVETs done among FSWs in SSA and reports a slightly lower retention than the 82% retention reported in another SiVET done among fisher folk in South-western Uganda [20]. HIV vaccine preparedness studies among other key populations outside SSA have reported higher retention than ours [21–23]. The retention we found could be explained by the fact that in our setting, FSWs are still criminalized, discriminated and encounter violence in the community [14, 15, 24], factors which may in turn hinder health care seeking and return for study follow up visits. They are also mobile [16] and this affects retention if study visits occur while they are out of the study area. We believe that amidst these challenges, the study team’s strategies such as phone call reminders, tracing volunteers for follow up visits and use of appointment cards as has been done elsewhere were vital in maintaining study visit attendance in one of the first SiVET studies to be done among FSWs in SSA. In addition, our retention results inform a vaccine trial scenario where data on vaccine efficacy and safety would only be meaningful if volunteers received all vaccinations and attended the last study visit for endpoint review.

### Factors associated with retention and vaccination completion

Studies of hepatitis B vaccination among FSWs and minority populations in South America have found that less than one third of volunteers complete the vaccination schedule [25, 26]. In our study women aged < 35 years were less likely to complete the vaccination schedule or be retained with the risk of non-retention being higher among the youngest age group (18–24 years). Similarly, in a study done among at-risk youth who were randomized to a standard 0, 1 and 6 months’ hepatitis B vaccination schedule versus an accelerated 0, 1 and 2 months’ schedule, younger volunteers were less likely to complete a vaccination schedule [27]. These findings are corroborated by work done in the HIV vaccine field including: HIV vaccine preparedness cohorts, and clinical trials of both vaginal microbicides and candidate HIV vaccines [28–30] and yet younger age has been associated with increased risk of HIV acquisition [31, 32]. In the broader context of HIV, studies of the “test and treat” intervention among HIV positive women from the same cohort as the SiVET study group [33] and elsewhere [34, 35] have found that younger people are less likely to initiate prompt anti-retroviral therapy (ART). Younger women from high risk communities are vulnerable and experience a greater degree of unequal power relations, gender inequities, exploitation, limited social support and intimate partner violence when compared to older women [36, 37] who also make the working environment uncomfortable for the younger ones by patronizing, bullying and exploiting them [36, 38]. This may affect their ability to engage with health services [39]. FSWs also work through pimps and middlemen, a group of stakeholders who usually control and make decisions for them, limit their independence [40, 41] and subsequently freedom to make the required study visits. The stakeholders are usually influential community members or those who hold power and vary by community. They include but are not limited to older FSWs, middlemen and community leaders, and have an important role in empowering younger women. Empowering young FSWs to participate more in HIV prevention programs as peer leaders and decision makers has been identified as a need [42] and may improve retention and vaccination completion in future HIV vaccine efficacy trials.

Volunteers who reported GUD in the past 3 months were less likely to complete the vaccination schedule or be retained. Studies among high risk women attending primary health care facilities have not found an association between STIs and vaccination completion [43], however elsewhere, STIs have been associated with Hepatitis B vaccination completion among MSM [44, 45]. Risk behavior such as injection drug use and multiple sexual partners have been associated with low completion of multi-dose vaccine schedules [25, 45]. GUD is a surrogate marker of risky sexual behavior yet low risk perception may lead to complacency with disease prevention and health promotion interventions; GUD has long been understood as a risk factor for HIV acquisition as well [46, 47]. Population based surveys done in different settings and age groups give mixed results on the association between risky sexual behavior and uptake of health interventions [48, 49]. Volunteers who report genital symptoms such as GUD need close follow up to ensure high retention in future trials.

Our finding that women who used condoms consistently with new sexual partners were less likely to complete the vaccination schedule, though unexpected, is similar to findings from two FSW cohort studies in Kenya and China that assessed attrition among HIV negative volunteers [50, 51]. On the contrary, Shokoohi et al. have reported inconsistent condom use among HIV negative women to be associated with a lower likelihood of recent HIV testing, a finding that is consistent with high risk behavior [52]. We believe that women may have felt protected by consistent condom use with new partners and were therefore less keen to attend study vaccination visits. Hepatitis B vaccine being used to simulate an HIV vaccine product may not have been perceived beneficial for HIV prevention like condoms which actually protect against HIV infection if used consistently. A survey of acceptability of future HIV vaccines has shown that acceptability increases if perceived benefits of the vaccine are higher [53]. Volunteers who report consistent condom use may miss vaccination visits in future HIV vaccine efficacy trials but also those who adhere to vaccination visits may engage in risk behavior such as inconsistent condom use or multiple sexual partnerships *(Risk Compensation)* as has been reported elsewhere [54, 55]. Volunteers who report consistent condom use need on-going education and counselling to enable vaccination completion in future HIV vaccine trials.

#### Limitations and strengths

This study used a licensed commercially available vaccine in lieu of an experimental product, thus observed outcomes may differ from what would be observed in a trial using a real HIV vaccine investigational product with unknown long-term safety profile. The SiVET enrolled from an existing cohort; the women who accept to get enrolled and access services in the general FSW cohort may be different from those who decline and remain in the community thereby introducing selection bias in the study sample and affecting generalizability of findings. The study procedures however were designed to mimic the rigors of an efficacy trial hence giving us more knowledge about feasibility of a future vaccine efficacy trial. Our retention findings are granular enough to provide information not only on last visit attendance but also completion of the vaccination schedule, an outcome important to demonstrate vaccine efficacy.

## Conclusions

FSWs in Kampala are at substantial risk of HIV infection and are willing to be enrolled in HIV vaccine and prevention research; however, younger FSWs may be harder to retain. There is a need to design strategies that overcome the social and community barriers faced by younger FSWs in order to improve participation in future vaccine efficacy trials. Such strategies would involve sensitization of influential and powerful community stakeholders about the need for young FSWs to participate in research and empowering the young FSWs to be decision makers in HIV prevention. Factors associated with high-risk behavior among FSWs such as STI symptoms also lead to lower retention and vaccination completion. Volunteers who report STI symptoms such as GUD need tracing not only for treatment but also for follow up to ensure high retention in future vaccine trials.

## Data Availability

The datasets used and analyzed during the current study are available from the corresponding author on reasonable request.

## Abbreviations

ART: Anti-retroviral treatment
FSWs: Female sex workers
GHWP: Good Health for Women Project
GUD: Genital ulcer Disease
HIV: Human Immunodeficiency Virus
HPTN: HIV Prevention Trials Network
HTC: HIV testing and counselling
HVTN: HIV Vaccine Trials Network
IAVI: International AIDS Vaccine Initiative
LSHTM: London School of Hygiene and Tropical Medicine
MRC: Medical Research Council
MRC/UVRI and LSHTM: Medical Research Council/ Uganda Virus Research Institute and London School of Hygiene and Tropical Medicine
MSM: Men who have sex with men
PrEP: Pre-exposure prophylaxis
SiVET: Simulated Vaccine Efficacy Trial
SSA: Sub-Saharan Africa
STIs: Sexually Transmitted Infections
UVRI: Uganda Virus Research Institute
VDS: Vaginal discharge syndrome
